# Editorial: Pathogenic Advances and Therapeutic Perspectives for Eosinophilic Inflammation

**DOI:** 10.3389/fmed.2018.00243

**Published:** 2018-08-31

**Authors:** Florence E. Roufosse, Mats W. Johansson

**Affiliations:** ^1^Department of Internal Medicine, Hôpital Erasme, Université Libre de Bruxelles, Brussels, Belgium; ^2^Department of Biomolecular Chemistry, University of Wisconsin, Madison, WI, United States

**Keywords:** eosinophils, interleukin-5, asthma, hypereosinophilic syndromes, eosinophilic esophagitis, mastocytosis

With the recent approval of the first eosinophil-depleting therapeutic agents targeting the Interleukin-5 (IL-5) pathway for treatment of severe eosinophilic asthma, eosinophils, and eosinophilic disorders are in the limelight. Setbacks during clinical development of these compounds have revealed how much remains to be known about eosinophil biology *in vivo*, and have nurtured profuse research both on basic eosinophil biology and on pathogenic disease mechanisms, in order to better delineate the most meaningful targets for innovative therapeutic strategies. On one hand, variable degrees of eosinophil depletion observed in some compartments during IL-5-targeted treatment indicate that certain eosinophil subsets may not rely on this cytokine and/or that other important pro-eosinophilic mediators and signaling pathways are operative *in vivo*. On the other hand, it is increasingly clear that disorders involving eosinophils such as asthma are the final outcome of complex interactions between diverse cell types and mediators, beyond eosinophils and IL-5. These include type 2 helper T (Th2) cells and innate lymphoid cells, mast cells, and a variety of factors that either activate eosinophils or are released by them. Although a considerable amount of research has focused on asthma because it is a common condition and because management of severe asthma remains a major challenge, several rare eosinophilic disorders with more homogenous features have proven to be extremely useful models to reach a better understanding of the involvement of eosinophils in tissue damage and dysfunction, and of the micro-environmental interactions operating within the complex network of eosinophilic inflammation. Unraveling this interplay has resulted in advances in the development of molecular tools to detect disease subsets and to monitor therapeutic responses, and in identification of promising new therapeutic targets. Precision medicine for management of eosinophilic disorders has now become a realistic endeavor.

This Research Topic dedicated to eosinophilic conditions comprises 26 articles, including reviews, mini-reviews, and perspective as well as hypothesis and theory articles. Their scope ranges from basic immunological research to clinically oriented topics, all chosen to stimulate curiosity and offer a wider and more comprehensive understanding of the numerous actors involved in these disorders. Translational aspects of research in the field of eosinophilic inflammation are highlighted throughout the Topic. Recent progress in the understanding of eosinophil biology and heterogeneity is reviewed, as well as insights into the contributions to and regulation of eosinophil trafficking and recruitment in asthma and other eosinophilic or allergic diseases by various families of mediators and their receptors, and by interactions with platelets. Eosinophil interactions with other lineages, including Th2 cells and mast cells, in inflammatory disorders are also addressed. Importantly, the Topic covers aspects of particular relevance for drug development, reporting on translational research investigating pathogenic mechanisms of specific eosinophilic disorders in humans, including asthma, that have greatly implemented modern classification of these disorders, and will likely result in significant changes in the way patients are managed through a more personalized approach to prognostication, prediction of treatment responses, and targeted therapy.

The first collection of papers presents the state-of-the-art in selected fields of basic eosinophil biology where significant progress has been made, including development, translation, and proteomics, offering a broad perspective on potential eosinophil-expressed targets. Fulkerson reviews insights into the mechanisms of gene regulation during eosinophil lineage commitment, differentiation, and maturation. She describes a model according to which classes of transcription factors (including GATAs, C/EBPs, PU.1, and XBP1) cooperate to direct eosinophil development and discusses the potential for therapeutic intervention. Accumulating evidence, reviewed by Johnston and Bryce, indicates that IL-33, usually considered an epithelial-derived cytokine that orchestrates allergic inflammation and contributes to type 2 immunity, together with its receptor ST2 also have roles in regulating eosinophil development. In addition, IL-33 is a potent activator of mature eosinophils. These advances should impact our understanding of how therapeutic targeting of this pathway may modulate disease. Esnault et al. provide an overview on protein translation within eosinophils, and its regulation by intracellular signaling. They address mRNA post-transcriptional regulation and focus on the role of IL-3, which, unlike the other IL-5 family cytokines IL-5 and GM-CSF, drives sustained signaling in eosinophils and increased translation of a subset of mRNAs, including semaphorin 7A and Fcγ receptor II (CD32), which may have clinical relevance in terms of eosinophil priming/activation *in vivo*. Further, they discuss mechanisms regulating mRNA-binding protein activity in eosinophils and the potential therapeutic targeting of these signaling pathways. As they indicate, mRNA and protein levels do not always correlate. Proteomic analysis of human eosinophils, using liquid chromatography coupled to tandem mass spectrometry, has recently identified and quantified >7,000 proteins and is reviewed by Mosher et al. They give examples of the power of such analysis to provide novel information on isoforms of proteins, including eosinophil STATs. Further, they describe how isobaric labeling has identified 220 phosphosites that change significantly upon acute eosinophil activation with IL-5. Finally, and importantly for this Research Topic, they discuss how these methods may prove valuable to address whether certain eosinophil proteins are altered or predict therapeutic outcomes in patients with eosinophilic diseases.

The next set of articles deals with the roles of eosinophils in asthma, regulation of eosinophil recruitment by multiple agonists and receptors, and eosinophil effector functions. McBrien and Menzies-Gow give a general overview of the eosinophil and of the current understanding of the (possible) roles of eosinophils in key asthma processes. These include evidence of eosinophil contributions to exacerbations and airway remodeling as well as mechanisms by which eosinophils may promote airway hyperresponsiveness and mucus secretion. In addition, the immunomodulatory roles of eosinophils are discussed. In this line, it is now well established that eosinophils are heterogeneous, with varying membrane-expressed receptors and secreted products. The characterization of homeostatic versus inflammatory eosinophils proposed by Marichal et al. in this Topic represents a major advance in understanding eosinophil heterogeneity and accounts for some of the intriguing findings made previously in the field of eosinophil research. Variable expression of the IL-5 receptor by certain homeostatic eosinophils is particularly relevant for treatment responses in anti-IL-5(R) treated patients. Larose et al. review eosinophil chemoattractants with an emphasis on eotaxins, other chemokines, and their receptors. Chemoattractants for type 2 innate lymphoid cells (ILC2s) are also evoked, emphasizing however that mechanisms for recruitment of these cells are relatively poorly defined so far. Although historically the focus has been on protein-protein interactions in biological systems, protein-carbohydrate interactions has recently received greater recognition. O'Sullivan et al. describe how lectin-glycan interactions can modulate eosinophil functions, including recruitment, survival, and inflammation. Their primary focus is on Siglec-8, expressed on human eosinophils, but selectins and their ligands, and other siglecs are also discussed. Finally, they consider potential therapeutic exploitation of these interactions in eosinophilic diseases, e.g., the Siglec-8 pathway in inducing cell death. Galectins, one of the lectin families, are expressed by various cells including eosinophils. Rao et al. review galectins that regulate eosinophil recruitment, activation, and apoptosis in allergic asthma, and are pro- (e.g., galectin-3, by interacting with α_4_ integrin) or anti-inflammatory (galectin-1). They discuss their potential utility as therapeutic targets. In addition, human but not murine eosinophils contain galectin-10 (Charcot-Leyden crystal protein), a potential biomarker for eosinophilic inflammation. Prostaglandins and leukotrienes are other families of molecules that can be pro- or anti-inflammatory. Peinhaupt et al. focus on prostaglandins (PG) D_2_ and E_2_, prostacyclin I_2_, and their receptors on eosinophils. PGD_2_ activates eosinophils, whereas PGE_2_ and I_2_ suppress activation. They summarize potential drug interventions, including antagonists of the PGD_2_ receptor DP2 (CRTH2). Such antagonists have produced improvements in lung function in subsets of asthmatic patients and some improvement in eosinophilic esophagitis (EoE). Thompson-Souza et al. review the cysteinyl leukotrienes LTC4, D4, and E4, which have various activities on eosinophils, in light of development of therapeutic compounds targeting their receptors. They discuss the two cysteinyl leukotriene receptors expressed by eosinophils pointing out that it has recently been recognized that they are also present and functional in the membrane of eosinophil free granules, raising the question whether free granules may be therapeutic targets beyond intact eosinophils. Related to eosinophil activation and recruitment, the activation status of eosinophils, as assessed by eosinophil surface proteins that are potential biomarkers, is described by Johansson. Circulating eosinophils may be non-activated or pre-activated (sensitized or “primed”) and their β_1_ integrin activation is associated with aspects of disease in non-severe asthma. β_2_ integrins on blood, but not airway, eosinophils, respond to intervention with anti-IL-5 mepolizumab. A model of eosinophil activation status in the circulation and the airway in asthma is presented; however the potential relevance of these biomarkers in eosinophilic diseases other than asthma requires future exploration. Tissue remodeling is a key feature of eosinophilic inflammation in a number of type 2 immune diseases. Nhu and Aceves review data and concepts on the pathogenesis of remodeling and fibrosis, primarily in EoE, including cytokines, eosinophils, and other immune cells, with relevant parallels in asthma. Additionally, they focus on how emerging therapies may reduce remodeling in a subset of patients.

Dealing with complex eosinophilic conditions is not achievable without considering the factors that interact with and/or are associated with eosinophilia. The following group of papers emphasizes some of the recent data and concepts relating to such factors. Like eosinophils, Th2 cells are heterogeneous, with pathogenic effector Th2 cells (peTh2) representing the most terminally differentiated subset, showing an exacerbated capacity to produce IL-5 in addition to IL-4 and IL-13, both produced earlier in the maturation process of Th2 cells. The phenotypic and functional characteristics of these cells are reviewed by Mitson-Salazar and Prussin, who argue that the critical role shown to be played by these cells in eosinophilic gastroenteritis may be operative in other eosinophilic conditions. Specific membrane-expressed molecules on these upstream inducers of eosinophilic inflammation may prove to be interesting targets for future therapeutic intervention. IL-13 is a cytokine involved in the pathogenesis of asthma and other type 2 immune conditions, eliciting mechanisms that promote eosinophil trafficking. Doran et al. provide a perspective on these roles of IL-13 in asthma and other eosinophilic disorders. They depict the IL-13 (and IL-4) receptors and antibodies blocking IL-13 or IL-13/IL-4 receptors, and describe the ongoing clinical trials with these antibodies. Another component interacting with eosinophils, e.g., in asthma, is the platelet. Although the importance of platelet activation during hemostasis is well understood, it is now also recognized that platelets can be activated and function in a distinct manner during inflammation; evidence indicates that they are critical in the pathogenesis of allergic diseases. Shah et al. explore non-thrombotic platelet activation in the context of allergy and the association of platelets with eosinophils, including conclusions drawn from platelet depletion experiments in animal models, as well as how these phenomena may yield novel therapeutic targets. The important interactions between eosinophils and mast cells, which are almost invariably present together in inflamed tissue (composing the “allergic effector unit”), are described in detail by Galdiero et al. in this Topic. In addition to the numerous direct interactions between these cells, possible Th2 cell-dependent indirect interactions may be relevant in eosinophilic disorders, all contributing to certain aspects of treatment responses. This paper closes the biological part of this Research Topic on eosinophilic conditions.

The final series of papers is clinically focused, and is meant to illustrate how translational research can contribute to improved understanding not only of disease mechanisms, but also of eosinophil functions and interactions. We begin with recent disease classifications incorporating molecularly or immunologically defined disease variants. The first two papers illustrate the complex and constantly evolving interplay between advances in pathogenic understanding and refinement of classification schemes. Current definitions and categories of asthma and hypereosinophilic syndromes are summarized by Svenningsen and Nair and Kahn et al., respectively, showing how they have been implemented in clinical practice and improved patient management considerably. However, their limitations are highlighted and perspectives for further amelioration are discussed. These limitations are mainly related to numerous gaps in our understanding of underlying pathogenic mechanisms, namely at the molecular level, with rigorous but empirical clinical observations supporting most current definitions. EoE has been chosen for this Topic as a model to show how rigorous application of disease-defining criteria has resulted in the constitution of a large and fairly homogenous collection of patients, paving the way to pathogenic breakthroughs that are likely to translate into major therapeutic advances in the coming years. Collins et al. describe the slow but rewarding process that has led to consensual determination of thresholds for tissue “hyper”-eosinophilia in different compartments of the digestive system. At the level of the esophagus, this has allowed for selection of patients for large-scale pathogenic studies. Elucidation of the transcriptome of EoE has delineated novel candidate targets for future drug development and has led to the development of the “EoE diagnostic panel” (EDP), the first application of a molecular approach to diagnosis in the setting of an allergic disorder. The EDP, described by Wen and Rothenberg herein, is now available for clinical use as a commercialized test. Its widespread use has revealed further heterogeneity within EoE, and it is hoped that this tool will allow for a personalized approach to future therapeutic decision-making, on the basis of specific molecular signatures in individual patients and the availability of an increasing number of targeted treatment options. Furthermore, genes whose transcription levels change with effective therapy may prove useful as future biomarkers of disease activity in eosinophilic disorders, for which there is much need. Khoury et al. provide an overview of the currently available biomarkers used to assess hypereosinophilic disorders, showing how improved understanding of pathogenesis (e.g., in EoE) has delivered the few robust markers that have been validated so far. For the majority of disorders, biomarkers enabling diagnosis of disease variants, and/or predicting disease severity and monitoring disease activity are lacking.

There is increasing interest for investigation of biomarkers in the setting of clinical trials with targeted therapy, in hopes of improving future selection of patients for tailored treatment. Two papers in this Topic review the use of monoclonal antibodies directed against IL-5 or its receptor and against IgE in eosinophilic disorders. Interestingly, elevated eosinophil counts are predictive of treatment response to both compounds in asthmatic subjects. This appears logical in that the cytokines responsible for induction of eosinophils and IgE production (i.e., IL-5 and IL-4/IL-13, respectively) are generally produced together by type 2 lymphocytes. Moreover, both eosinophils and IgE contribute jointly to inflammation and disease manifestations, and eosinophils express IgE receptors, so it is legitimate to explore how these factors interact *in vivo* in treated subjects. Roufosse reviews clinical trials targeting the IL-5 pathway and summarizes transversal data across these studies on biomarkers that may predict treatment responses, and on how other mediators and cell types, namely mast cells, are impacted by treatment. This paper also describes how the slow but determined development of anti-IL-5 antibodies, with careful *post-hoc* assessment of data collected during clinical trials, has resulted in a better understanding of pathogenic mechanisms underlying specific aspects of diseases under study, leading to improved design of subsequent trials, and ultimately, approval of new first-in-class drugs. Stokes reviews the studies evaluating efficacy of anti-IgE treatment, showing that eosinophilia decreases during treatment through mechanisms that remain largely unexplored. Some of the favorable effects of anti-IgE treatment on allergic disease may therefore actually be related to indirect effects on eosinophils, either through their depletion or their decreased priming *in vivo*. The article by Vaes et al. approaches mast cell targeted therapy and has been included in this Topic to provide insight on the different levels at which therapeutic intervention is possible in a hematological disorder where, like in hypereosinophilic syndromes, cell-mediated toxicity is often more of a concern than tumor burden. Drug development in mastocytosis and related disorders is very broad, including molecular targets, signaling machinery, mediator interception, and apoptosis, and may inspire new avenues of thought for predominantly eosinophilic diseases. Finally, the interesting pathogenic complexity of a secondary hypereosinophilic disorder, drug-reaction-with-eosinophilia-and-systemic-symptoms (DRESS), is reviewed by Musette and Janela. The combined existence of genetic predisposition, environmental exposure to an offending agent (a drug), and viral reactivation concurs toward the rapid development of marked blood and tissue eosinophilia, contributing to vital organ damage and death in some cases. The uncontrolled immune activation involves not only eosinophils but also cytotoxic CD8 T cells whose pathogenic contribution to other eosinophilic conditions has barely been explored and may be largely underestimated.

To conclude, we are very grateful to our colleagues including researchers, physicians, and clinical investigators who have contributed to this Research Topic. We believe that these articles offer an interesting and translational perspective on basic eosinophil biology, with clinical applications in diagnosis and treatment of eosinophilic conditions. They illustrate particularly well how combined experimental and clinical efforts to break down heterogeneous human diseases into mechanistic subsets can be rewarding and can translate into major improvements in patient management and outcome. We hope the contents of this Topic will further stimulate transversal thinking in the exciting field covered by this e-book.

## Author contributions

FR and MJ designed and drafted this manuscript, revised it, and approved the final version.

### Conflict of interest statement

FR has served as consultant for GlaxoSmithKline and Knopp Biosciences for clinical trial design in hypereosinophilic syndromes. MJ has received a fee for consulting from Guidepoint Global, a fee from Genentech for speaking, and funds for research from Hoffmann-La Roche; and has been an advisory board member for Genentech.

